# Naringenin ameliorates swine pulpitis by modulating immune response

**DOI:** 10.1186/s12903-026-08296-5

**Published:** 2026-04-14

**Authors:** Qian Wang, Wenfeng Zeng, Huilin Liang, Syngcuk Kim, Lanting Shao, Yan Yan, Yumeng Guo, Siqi Huang, Ying Zheng

**Affiliations:** 1 School of Stomatology, Capital Medical University, Beijing, China; 2 Department of Stomatology, Peking Union Medical College Hospital, Chinese Academy of Medical Sciences & Peking Union Medical College, Beijing, China; 3 Key Laboratory of Biomacromolecules, Institute of Biophysics, Chinese Academy of Sciences, Beijing, China; 4 Department of Pharmacy, China Pharmaceutical University, Nanjing, China; 5 Department of Endodontics, School of Dental Medicine, University of Pennsylvania, Philadelphia, PA USA

**Keywords:** Naringenin, Pulpitis, Dentin-like tissue regeneration, Neutrophils, TFEB-mediated lysosomal activity

## Abstract

**Background:**

Preserving dental pulp vitality is crucial for maintaining the physiological function of the tooth. Naringenin (Nar), known for its immunomodulatory properties, has shown pharmacological effects on various inflammatory diseases. This study aimed to investigate the therapeutic potential of Nar in treating *Porphyromonas gingivalis* lipopolysaccharide (*P.g*-LPS)-induced pulpitis in a swine model and to elucidate the mechanisms underlying its therapeutic efficacy.

**Methods:**

Swine premolars with *P.g*-LPS-induced pulpitis were divided into four groups: sham, hydrogel, iRoot BP PLUS, and Nar hydrogel. Treatment outcomes were evaluated by assessing neutrophil infiltration, dentin-like tissue regeneration, and coronal pulp tissue preservation using histological and immunohistochemical techniques. To further examine potential cellular responses, the effects of Nar were studied in human dental pulp fibroblasts (hDPFs), human peripheral blood-derived neutrophils (hNeu.), and differentiated HL-60 cells (dHL-60). Surface markers were analyzed using fluorescence-activated cell sorting (FACS), cytokine levels were measured by ELISA, and mineralization was assessed using alkaline phosphatase and Alizarin Red S staining. Neutrophil phagocytosis, bactericidal activity, intracellular reactive oxygen species (ROS) levels, and translocation of transcription factor EB (TFEB)-mediated lysosomal activity were evaluated. Statistical analyses included Shapiro-Wilk test and one-way ANOVA or Kruskal-Wallis test with appropriate post hoc comparisons.

**Results:**

The sham group showed severe pulp tissue necrosis and an intense inflammatory response. Hydrogel alone exhibited limited therapeutic effects. Both Nar hydrogel and iRoot BP PLUS promoted dentin-like tissue formation; however, Nar hydrogel reduced inflammatory infiltration and preserved a greater proportion of coronal pulp tissue. In vitro, Nar reduced inflammatory cytokine secretion from hDPFs and neutrophils and improved *P.g*-LPS-impaired mineralization capacity in hDPFs. Nar also enhanced the phagocytic and bactericidal activities of dHL-60 cells, accompanied by controlled ROS elevation and increased TFEB-mediated lysosomal activity.

**Conclusions:**

Nar demonstrates therapeutic potential for pulpitis management by modulating inflammation and promoting dentin-like tissue regeneration. Its efficacy likely stems from the fine-tuning of inflammatory responses. These findings suggest that Nar may represent a potential biological alternative to conventional pulp capping materials for vital pulp therapy.

**Supplementary Information:**

The online version contains supplementary material available at 10.1186/s12903-026-08296-5.

## Background

Pulpitis is one of the most common diseases in oral medicine and is primarily caused by bacterial invasion and infection [1]. Early-stage pulpitis is characterized by pulp congestion, inflammation, nociceptor activation, and severe pain [2]. As the condition progresses, ischemia and necrosis of pulp tissue ensue, often culminating in root canal treatment as the final therapeutic option [3–5]. Clinically, vital pulp therapy (VPT) is widely adopted to preserve pulp vitality [6]. Although calcium silicate-based materials have shown favorable outcomes in vital pulp procedures, their performance in inflamed pulp tissue exhibits a higher failure rate [7]. Therefore, developing strategies that can regulate the inflammatory microenvironment is of critically important for treating pulpitis. Ideally, pulpitis treatment should aim to eliminate infection, attenuate inflammation, maintain pulp vitality, and optimize the regeneration of dentin-pulp complex.

Pathological studies of caries-derived pulpitis indicate that the vast majority of bacteria are localized in the carious dentin and necrotic tissue, with only a small number of bacteria present in the inflamed pulp beneath the necrosis [8, 9]. The intensity and duration of the secondary inflammatory responses and circulatory disorders determine the outcome of the pulpitis [10]. Recent research emphasizes the roles of neutrophils and dental pulp fibroblasts (DPFs) in pulp inflammation and mineralization processes [9, 11]. These cells and their associated factors show complex and diverse functions. Neutrophils effectively eliminate pathogenic bacteria primarily through the production of reactive oxygen species (ROS), which serve as a central antimicrobial mechanism of innate immunity [12]. However, excessive ROS generation may lead to collateral tissue damage and necrosis [13]. Persistent neutrophil accumulation and activation can exacerbate hypoxia [14], leading to lysis and necrosis of healthy pulp tissue [15–17] and causing hemodynamic disturbances that may further impede the natural healing of the inflamed pulp. DPFs, the most abundant and widely distributed cell population in pulp tissue, play a key role in defense and repair by responding swiftly to external stimuli and forming mineralized nodules [18, 19]. However, elevated levels of inflammatory cytokines such as TNF-α, IL-6, and IL-1β impair the mineralization capacity of DPFs [20], thereby hindering the repair and regeneration of inflamed pulp tissue. Collectively, these findings suggest that modulating pulp inflammation to an optimal level may represent the most effective therapeutic strategy for pulpitis.

Given the central role of ROS in neutrophil-mediated tissue injury, naringenin (Nar), a natural flavanone compound (C_15_H_12_O_5_), has been reported to possess anti-inflammatory, ROS-modulating, immunomodulatory, and vasodilatory properties [21–23]. These properties suggest that Nar may help mitigate neutrophil-induced oxidative damage without compromising bactericidal activity. In animal models, Nar has demonstrated therapeutic potential as an adjunct in treating infections, cardiovascular diseases, and tumors [24]. Furthermore, Nar has been shown to promote the mineralization of human dental pulp stem cells [25, 26], supporting its potential role in repairing inflamed pulp tissue.

A thermoresponsive Pluronic F-127 (PF-127) hydrogel system was used to aid local delivery of Nar. The micellar structure of PF-127, combined with PEG200 as a co-solvent, enhances the solubility of Nar. This system remains flowable at 4 °C for precise administration and gels rapidly at body temperature [27]. Furthermore, its potential barrier effect [28] may stabilize the local microenvironment, supporting dentin-pulp complex regeneration.

We hypothesized that Nar could modulate inflammation and promote dentin-like tissue formation in pulpitis. To test this hypothesis, we used a swine model that resembles human teeth and pulpal healing dynamics [29, 30]. Additionally, we explored the potential mechanisms of Nar through in vitro experiments.

## Materials and methods

### Preparation of naringenin hydrogel

Naringenin (CAS 67604-48-2), a light brown powder with 98.5% purity (HPLC), was obtained from Sigma-Aldrich (Lot# W530098). Naringenin (Nar) hydrogel, generously provided by the Institute of Biophysics, Chinese Academy of Sciences (CAS), was prepared following a previously described protocol [27]. Briefly, Nar-PEG200 dissolution followed by dropwise addition to 25% (w/v) PF-127 at 4 °C and sterilization protocol (0.22 μm filtration) to ensure full reproducibility. The hydrogel, containing 2 mmol/L of Nar, remained flowable at 4 °C, and solidified at temperatures exceeding 19 °C.

### Experimental animals and study design

Eight Bama miniature swine (aged 18–20 months, weighing 40–50 kg) with permanent dentition were sourced from Beijing Shi Chuang Century Mini-pig Breeding Base and were acclimated for at least 1 week in the animal house of Capital Medical University. All swine were housed under conventional conditions, including clean and disinfected rooms, optimal ventilation and temperature, strict biosecurity measures, ad libitum access to water, and a standard soft food diet.

Two swine (12 premolars) were used to establish and validate the pulpitis model. The remaining six swine contributed 44 premolars for the therapeutic comparisons. The 44 premolars were randomly allocated to the experimental groups using a split-mouth design to minimize anatomical and inter-animal variability. Each premolar was treated as an experimental unit for analysis. In all groups, the treated teeth were sealed consistently to ensure uniformity across groups. Teeth that were structurally damaged during extraction (e.g., root fracture) were excluded from further analysis.

At eight weeks postoperatively, swine were humanely euthanized by intravenous injection of sodium pentobarbital (150 mg/kg), in accordance with the ARRIVE 2.0 and the American Veterinary Medical Association (AVMA) Guidelines for the Euthanasia of Animals (2020). Death was confirmed by the absence of heartbeat and corneal reflex. A comprehensive evaluation of treated teeth was subsequently conducted. Humane endpoints, including significant weight loss, lethargy, vocalization, reluctance to move, reduced appetite, or signs of distress, were monitored. None of the animals reached these endpoints during the study.

### Induction of pulpitis

A total of 56 healthy premolars (P2-P4) in both the maxilla and mandible were selected as experimental teeth. Inclusion criteria required that premolars be fully erupted, exhibit normal morphology, and show no evidence of dental caries or periodontal disease. General anesthesia was administered using ketamine chloride (6 mg/kg) and xylazine (0.6 mg/kg), following standard protocols. To induce pulpitis, a 2 mm diameter exposure was created in the selected teeth. Subsequently, a sterile cotton ball soaked in 10 µL of *P.g*-LPS (InvivoGen, USA) at a concentration of 10 mg/mL was applied to the pulp exposure site for 5 min. After removal of the cotton ball, a sterile gelatin sponge (~ 2 mm thick) was placed over the exposure. The cavity was then sealed with a glass ionomer cement (GIC; GC Fuji IX GP, Japan) for 7 days. Twelve premolars from two swine were harvested after 7 days to evaluate the success rate of pulpitis development and to assess early pulp inflammatory responses.

### Treatment procedures

The study design, including group allocation and experimental unit definition, was determined prior to the intervention phase. Sham group (*n* = 8 teeth): After 7 days of pulpitis model preparation, the closed pulp cavities were reopened exposing the pulp, and the cavities were sealed directly with GIC; iRoot BP PLUS group (*n* = 14 teeth): After pulp exposure, iRoot BP PLUS (Innovative Bioceramix, Vancouver, Canada) was applied as an intermediate restorative layer, then sealed with GIC; Hydrogel group (*n* = 8 teeth): A hydrogel (20 µL) was dispensed onto the pulp exposure site as an intermediate layer and subsequently sealed with GIC; Naringenin (Nar) Hydrogel group (*n* = 14 teeth): Similarly, 20 µL of Nar hydrogel was applied to the exposure site as an intermediate layer, followed by seal.

### In vivo evaluation

#### Histological and immunohistochemical evaluation

Histological examinations included hematoxylin and eosin (H&E) staining to evaluate pulp tissue morphology, immunohistochemical (IHC) staining for myeloperoxidase (MPO) to assess neutrophil infiltration [31], high mobility group box 1 (HMGB1) to detect necrosis, dentin sialophosphoprotein (DSPP) to identify odontoblast-like cells, and goldner trichrome staining to observe mineralized tissue formation and nuclear polarity. The extent of inflammatory infiltration was evaluated according to the inflammation grading method as described in previous study [32]. The inflammation extensity in the pulp was graded as follows: absent (score 1), mild (inflammatory cells next to dentin bridge or area of pulp exposure only, score 2), moderate (inflammatory cells observed in one third or more of the coronal pulp or in the midpulp, score 3), severe (all of the coronal pulp is infiltrated or necrotic, or the entire pulp is necrotic). Details of IHC antibodies were provided in the Additional file 1. Negative controls were performed by omitting the primary antibody under identical experimental conditions. Representative negative control images are shown in Supplementary Fig. S1a-c [see Additional file 1]. To ensure unbiased analysis, slides were coded and analyzed by two experienced histopathologists blinded to group assignments.

### Cell isolation and identification

#### Immunomagnetic cell sorting and cell identification

The isolation and culture of human dental pulp fibroblasts (hDPFs) and human peripheral blood-derived neutrophils (hNeu.) were conducted in accordance with protocols approved by the Ethics Committee of Beijing Stomatological Hospital, Capital Medical University (CMUSH-IRB-KJ-PJ-2023-19), and written informed consent was received prior to participation. Human dental fibroblasts (hDPFs) were isolated through magnetic-activated cell sorting (MACS) method (130-050-601, Miltenyi Biotec) based on previous protocol [33]. Human neutrophils (hNeu.) were isolated from peripheral blood using MACS (130-104-434, Miltenyi Biotec) following the manufacturer’s instructions.

hDPFs were cultured in α-minimal essential medium (MEM-α, Gibco) supplemented with 10% heat-inactivated fetal bovine serum (FBS, Gibco) and 1% penicillin/streptomycin (Beyotime) at 37 °C in a humidified atmosphere of 5% CO_2_. Cell surface markers, including CD44, CD105, CD90, CD146, STRO-1, CD34, and CD45, were identified using fluorescence-activated cell sorting (FACS).

Human neutrophils were maintained in RPMI 1640 medium (VivaCell) supplemented with 10% heat-inactivated FBS and 1% penicillin/streptomycin. FACS analysis of CD45, CD16, and CD66b was used for cell identification.

Antibodies used for ICC and FACS were detailed in the Additional file 1.

#### Immunocytochemical staining

Immunocytochemical staining for vimentin was conducted to identified the hDPFs [34]. hDPFs were seeded on coverslips and fixed with 4% paraformaldehyde. Following permeabilization with 0.5% Triton X-100 and blocking by 3% bovine serum albumin (BSA, NOVON SCIENTIFIC), cells were incubated with rabbit anti-vimentin polyclonal antibody overnight at 4 °C. Detection was performed using HRP-conjugated goat anti-rabbit IgG (H + L) and visualized with DAB staining. A negative control was prepared by omitting the primary antibody under identical experimental conditions, and a representative image is shown in Supplementary Fig. S1d [see Additional file 1].

#### Induction and characterization of differentiated HL-60 neutrophil-like cells (dHL-60)

The HL-60 cell line (CTL-240, ATCC) was cultured in Iscove’s Modified Dulbecco’s Medium (IMDM, Gibco) supplemented with 20% heat-inactivated FBS and 1% penicillin/streptomycin. Differentiation into neutrophil-like cells (dHL-60) was achieved using 1.3% dimethyl sulfoxide (DMSO, Sigma-Aldrich) for 6–7 days as previously described [35]. Differentiation was confirmed by FACS analysis using anti-human CD11b antibody (Supplementary Table S1, [see Additional file 1]).

### In vitro functional assays

#### Cell viability assay

Cell viability was assessed using CCK8 assays (LABLEAD) following 24-hour treatment with varying concentrations of naringenin (Nar, Sigma-Aldrich).

#### Inflammatory cytokine detection

hDPFs, hNeu., and dHL-60 cells were treated with different concentrations of Nar in the presence or absence of 1 µg/mL *P.g*-LPS for 24 h. Cytokine levels (IL-6 and IL-8) in the culture supernatants were quantified using ELISA kits (DY206-05 and DY208-05, R&D Systems). Absorbance was measured at 450 nm using a microplate reader (MK30, ThermoFisher Scientific).

#### Mineralization induction and assessment

hDPFs were plated into 6-well plates (Corning) at a density of 1 × 10^5^ cells/well, and cultured in two different media: regular medium (MEM-α), and mineralization-inducing medium (MM) as previously described [26].

To assess the effects of dHL-60 supernatants on hDPF mineralization, supernatants were collected from four groups of dHL-60 cells: untreated cells, cells treated with 1 µg/mL *P.g*-LPS, cells treated with 1 µg/mL *P.g*-LPS and 25 µmol/L Nar, and cells treated with 1 µg/mL *P.g*-LPS and 100 µmol/L Nar. Supernatants were harvested after 24 h, mixed with MM in a 1:1 ratio (v/v), and applied to hDPFs.

To evaluate the direct effects of Nar on hDPF mineralization, cells were cultured in MEM-α, MM, or MM supplemented with 1 µg/mL *P.g*-LPS, in the presence or absence of 25, 100 µmol/L Nar.

Media in all groups were refreshed every three days. Alkaline phosphatase staining was conducted after 7 days, and Alizarin Red S staining was performed after 21 days.

##### Alkaline phosphatase staining

To detect the expression of alkaline phosphatase, the BCIP/NBT alkaline phosphatase colour development kit (Beyotime Biotech Co, Shanghai, China) was applied.

##### Alizarin red S staining

Alizarin red S staining was conducted following established protocols [26, 36]. Briefly, cells were stained with 2% Alizarin Red S (Sigma-Aldrich) at RT for 40 min. Mineralized deposition was visualized using a light microscope (ECLIPSE E100, Nikon).

#### Neutrophil functional assays

##### Intracellular ROS measurement

dHL-60 cells were loaded with 2’,7’-dichlorofluorescin diacetate (DCFH-DA) in serum-free IMDM medium for 20 min at 37 °C, following the instructions of Reactive Oxygen Species Assay Kit (S0033S, Beyotime). After treatment with 1 µg/mL *P.g*-LPS or varying concentrations of Nar for 1 h, the mean fluorescence intensity (MFI) was measured by the FACS analysis (Calibur, BD Biosciences).

##### Phagocytosis

Phagocytic assay was evaluated using a slightly modified protocol [37]. dHL-60 cells were pretreated with 25 µmol/L Nar with or without 4 mmol/L N-acetylcysteine (NAC, Beyotime) for 1 h, followed by incubation with enhanced green fluorescent protein (EGFP)-labeled *E. coli* (kindly provided by Prof. Ping Zhu, Institute of Biophysics, CAS) at a multiplicity of infection (MOI) of 10 in antibiotic-free IMDM medium for 15 min at 37 °C with rotation at 100 rpm. After incubation, cells were washed and analyzed by FACS.

##### Bactericidal assay

The protocol for bactericidal assays is slightly modified from the previous study [37]. dHL-60 cells were treated as described in *Phagocytosis* section. These cells were exposed to *E. coli* for 2 h. Then, cells were lysed in 1% Triton X-100, and lysates were diluted and spread on LB agar plates. Colony-forming units (CFU) were counted after overnight incubation at 37 °C.

### Molecular and imaging analyses

#### Cell transfection

Cell transfection was performed when HEK293T cell reached 50–60% confluence. The DNA-PEI complex was prepared by mixing the pEGFP-N1-TFEB plasmid with PEI 40000 (40816ES02, Yeasen). Subsequently, 100 µL of the DNA-PEI complex was added dropwise to each dish, gently mixed, and incubated for 36–48 h.

#### Confocal microscopy

dHL-60 cells were treated with 25 µmol/L Nar for 1 h, followed by incubation with EGFP-labeled *E. coli* (MOI = 10) for 15 min at 37 °C. Cells were washed with PBS (pH 7.4) and stained with 75 nmol/L LysoTracker Red DND-99 (L7528, Invitrogen) for 15 min. After washing, cells were placed in a live cell imaging chamber maintained at 37 °C with 5% CO_2_ and 95% humidity. Lysosomes, *E. coli*, and their colocalization were visualized using a laser scanning confocal microscope (LSCM, FV3000RS, Olympus, Japan).

To analyze the intracellular distribution of TFEB, HEK293T cells expressing pEGFP-N1-TFEB were pretreated with or without 4 mmol/L NAC, in the presence of different concentrations of Nar (0, 25, 100 µmol/L) for 1 h. TFEB and nuclear localization were visualized using FV3000RS LSCM after staining the nuclei with Hoechst 33342 (C1029, Beyotime) for 10 min.

Images were randomly selected from at least 3 different regions per group and analyzed using ImageJ software (National Institute of Health, Bethesda, MD, USA).

#### Lysosome quantification

For flow cytometry assays, dHL-60 cells were treated as described in *confocal microscopy* section, washed with PBS (pH 7.4), and then stained with 75 nmol/L LysoTracker Green DND-26 (C1047S, Beyotime) for 15 min. Lysosomal numbers were assessed by measuring green fluorescence using the FACS analysis (Calibur, BD Biosciences). Under LSCM, lysosomal numbers were quantified by measuring the mean gray value of red fluorescence stained by LysoTracker Red DND-99. Phagocytic and lysosomal digestive abilities were evaluated by analyzing the colocalization of lysosomes and EGFP-labeled *E. coli.*

### Statistical analysis

In vitro experiments were performed in at least triplicate, with a minimum of three replicates per condition. For the in vivo experiments, a formal a priori sample size calculation was not performed. The sample size was determined prior to the intervention phase based on previous swine studies of pulp biology [30, 38], and the Resource Equation approach for animal experiments. In the final one-way analysis of variance (ANOVA) analyses, the error degrees of freedom (E; calculated as total experimental units minus the number of groups) exceeded 20. Statistical analyses were conducted using GraphPad Prism 9 software. Data are presented as mean ± standard deviation (SD). Normality was assessed using the Shapiro-Wilk test. Homogeneity of variance was evaluated using the Brown-Forsythe test. For normally distributed data with equal variances, comparisons between two groups were performed using unpaired two-tailed *t*-tests, and comparisons among multiple groups were conducted using one-way ANOVA followed by Tukey’s multiple comparisons test. For normally distributed data with unequal variances, comparisons among multiple groups were performed using Brown-Forsythe and Welch ANOVA, followed by Dunnett *T*3 multiple comparisons test. For non-normally distributed data, the Kruskal-Wallis test with Dunn’s multiple comparisons test was used. Statistical significance was defined as *P* < 0.05.

## Results

### Histopathological evaluation of the pulpitis model

**Fig. 1:**
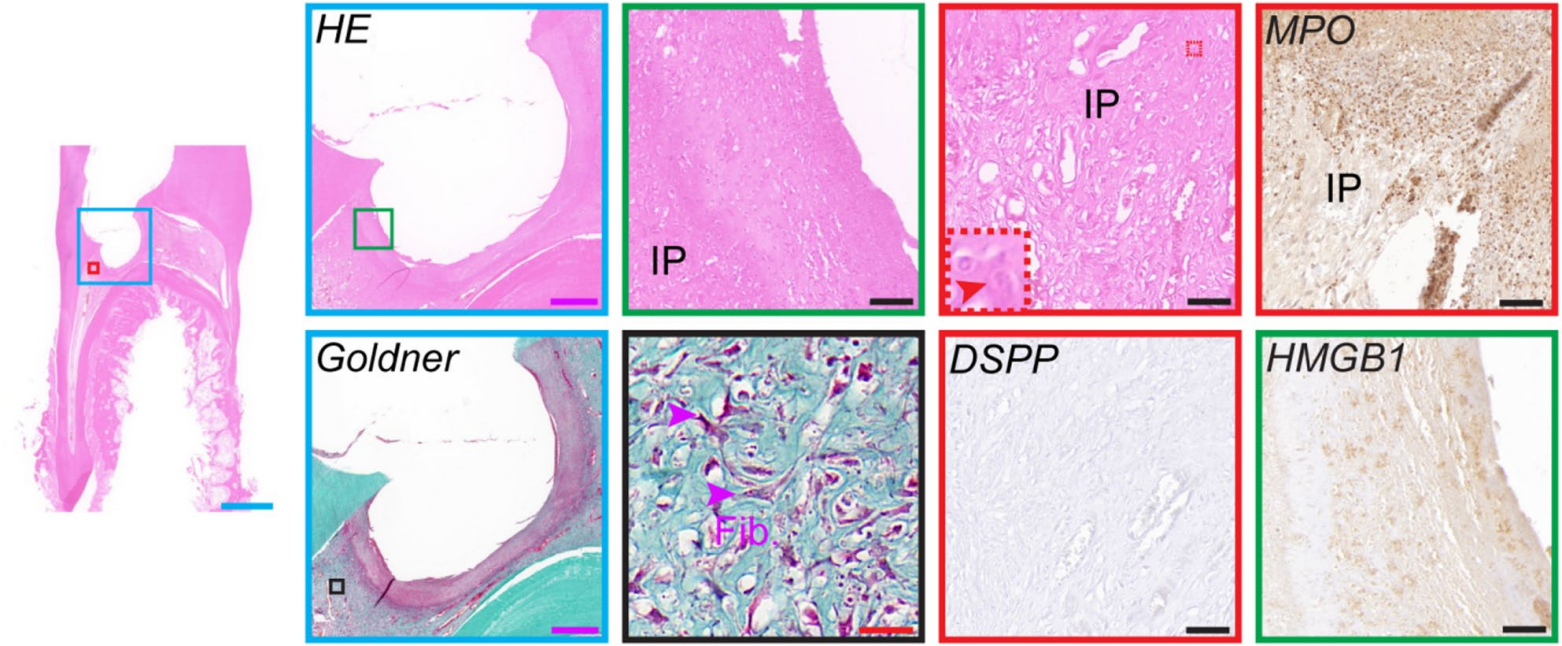
Histopathological assessments of pulpitis model in swine. Red arrow indicate to neutrophils, purple arrows indicate to dental pulp fibroblasts (Fib.). IP, inflamed pulp. Scale bars: 2 mm (blue), 500 μm (purple), 50 μm (black), 20 μm (red)

Seven days after *P.g*-LPS induction, necrosis and inflammatory cell infiltration was observed around the pulp exposure site (Fig. 1). A distinct necrotic band was identified, characterized by curled and easily detachable necrotic tissue that separated from the surrounding inflamed pulp and lacked discernible cellular morphology. Neutrophils were prominently observed in the inflamed coronal pulp and were identified by lobulated nuclei on H&E staining (red arrow in Fig. 1) and MPO-positive immunostaining. Goldner trichrome staining further delineated the necrotic zone, and the inflamed pulp beneath predominantly consisted of elongated, spindle-shaped, or polygonal dental pulp fibroblasts (purple arrows in Fig. 1). DSPP-positive cells were absent. HMGB1-positive staining validated the necrotic zone. Among 12 samples, 10 teeth showed severe pulpitis, manifesting as extensive neutrophil infiltration and tissue necrosis, whereas the remaining 2 teeth exhibited moderate pulpitis with neutrophil infiltration comprising at least one-third of the coronal pulp. The success rate of the severe pulpitis model was 83.3%.

### Histopathological evaluation of experimental groups at eight weeks

Of the 44 premolars initially allocated to the experimental groups, four were excluded from analysis due to root fracture during extraction. The final sample sizes were 8 in the sham group, 8 in the hydrogel group, 12 in the iRoot BP PLUS group, and 12 in the Nar hydrogel group.

**Fig. 2:**
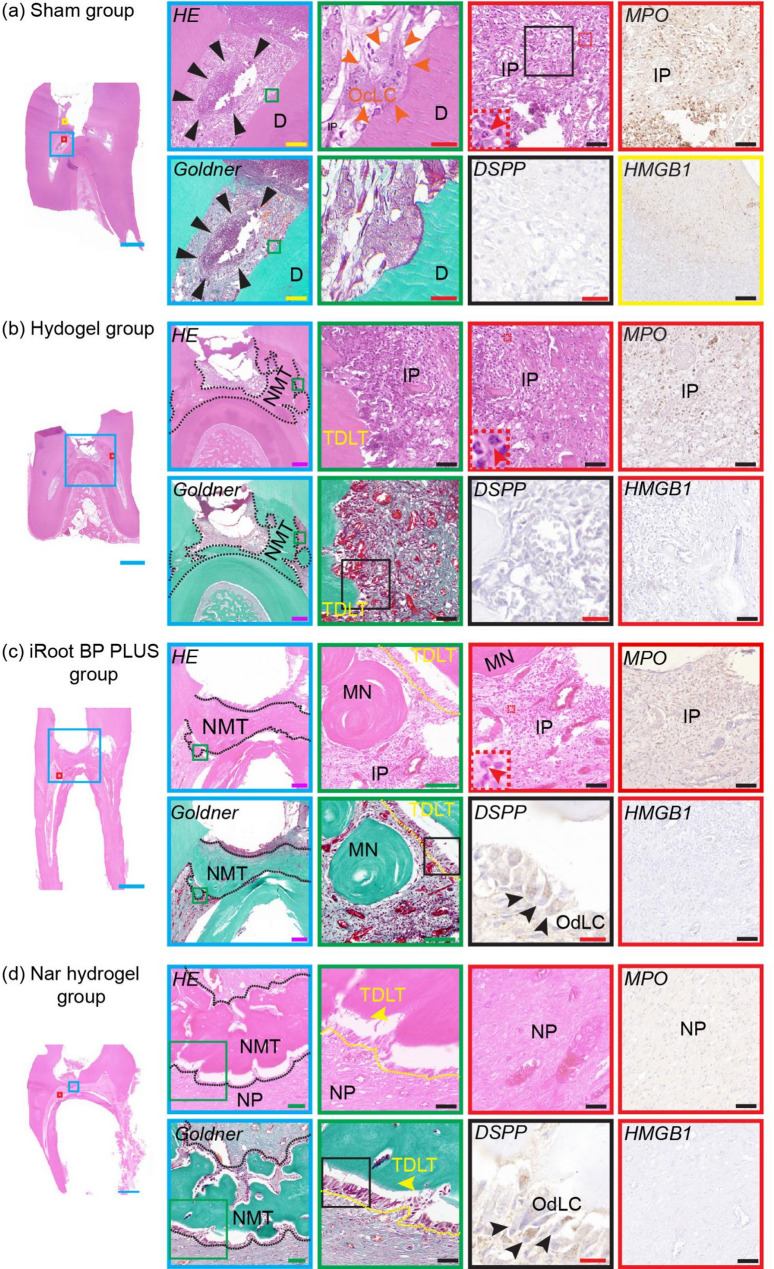
Histopathological evaluation of experimental groups at eight weeks. Histopathological assessments were conducted in (**a**) sham group, (**b**) hydrogel group, (**c**) iRoot BP PLUS group, and (**d**) Nar hydrogel group. Black triangles indicate to necrosis, orange arrows indicate to odontoclast-like cells (OcLC), red arrows indicate to neutrophils, black arrows indicate to odontoblast-like cells (ObLC), yellow arrows indicate to tubular dentin-like tissue (TDLT), black dashed lines draw the extension of newly formed mineralization, and yellow dashed lines indicate the odontoblast-like cell layer. IP, inflamed pulp; NP, normal pulp; MN, mineralized nodules; D, dentin; NMT, newly formed mineralized tissue. Scale bars: 2 mm (blue), 500 μm (purple), 200 μm (yellow), 100 μm (green), 50 μm (black), 20 μm (red)

In the sham group, severe inflammation was observed, characterized by tissue disintegration and extensive neutrophil infiltration within the root pulp (Fig. 2a). The central zone of liquefactive necrosis was densely packed with MPO-positive neutrophils. H&E and Goldner trichrome staining showed internal resorption of the root canal wall, accompanied by multinucleated odontoclast-like cells on the resorptive lacunae (orange arrows in Fig. 2a). DSPP-positive areas were absent. HMGB1-positive staining confirmed necrosis at the exposure site. These histological features indicate progressive pulp tissue deterioration in the absence of treatment.

In the hydrogel group, mineralized tissue formation was observed within the coronal pulp space (Fig. 2b). Newly formed dentin-like tissue extended to the root canal orifices, with calcified globules within the root pulp. Numerous inflammatory cells, including MPO-positive neutrophils and chronic inflammatory cells, were observed in the pulp tissue adjacent to the newly formed tubular dentin-like tissue. Goldner trichrome staining confirmed the mineralized tissue formation, which was accompanied by vascular dilation and inflammatory cell infiltration. No necrosis was detected histologically (HMGB1-negative), and DSPP-positive cells were absent.

In the iRoot BP PLUS group, mineralized tissue formation was observed, accompanied by moderate inflammatory cell infiltration (Fig. 2c). A thick layer of newly formed dentin bridge occupied a considerable portion of the coronal space, leaving only a thin layer of residual soft pulp tissue. Tubular dentin-like tissue and an odontoblast-like cell layer were present. However, irregular scattered mineralized nodules, numerous inflammatory cells (neutrophils and chronic inflammatory cells), and dilated blood vessels were also observed beneath the newly formed dentin bridge. Goldner trichrome staining confirmed these findings and highlighted inflammatory cells beneath the odontoblast-like cell layer (yellow dashed line in Fig. 2c). The odontoblast-like cells were DSPP-positive. No histologically detectable necrosis was observed.

In contrast, the Nar hydrogel group showed largely preserved pulp architecture with minimal inflammatory cell infiltration (Fig. 2d). A continuous dentin bridge was formed at the pulp exposure site, leaving an adequate soft pulp tissue. MPO staining revealed no evident neutrophil infiltration. The root pulp tissue exhibited no obvious pathological alterations. Beneath the newly formed tubular dentin, a distinct odontoblast-like cell layer (yellow dashed lines in Fig. 2d) and normal-appearing coronal pulp were observed. DSPP staining identified odontoblast-like cells. HMGB1 staining showed no histologically detectable necrosis. These findings indicate reduced inflammatory features and preservation of pulp tissue architecture in the Nar hydrogel group.

**Fig. 3:**
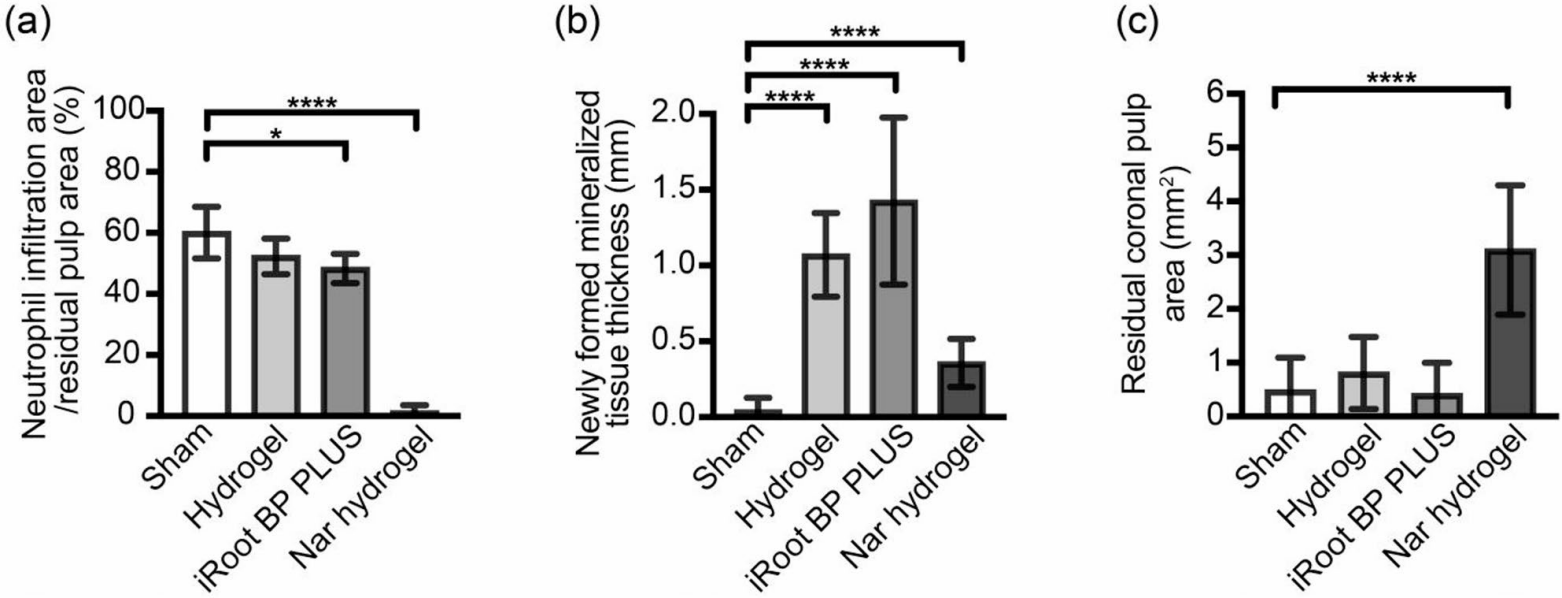
Semi-quantitative histomorphometric analysis of the experimental groups. **a** Percentage of neutrophil infiltration in the residual pulp area. **b** Thickness of newly formed mineralized tissue. **c** Residual coronal pulp area. Of the 44 premolars initially allocated to the therapeutic groups, four were excluded due to root fracture during extraction. Final sample sizes were *n* = 8 for the sham group, *n* = 8 for the hydrogel group, *n* = 12 for the iRoot BP PLUS group, and *n* = 12 for the Nar hydrogel group. Data are shown as mean ± SD.****P < 0.0001,*P < 0.05

The quantification of inflammation extent is shown in Table 1. The number of inflammatory cells in the Nar hydrogel group was negligible and significantly lower than that in the sham, hydrogel, and iRoot BP PLUS groups (*p* < 0.0001, Fig. 3a). Although the iRoot BP PLUS and hydrogel groups showed greater mineralized bridge thickness than the Nar hydrogel group (Fig. 3b), the Nar hydrogel group preserved a larger portion of the coronal pulp tissue (Fig. 3c).

**Table 1:** Inflammatory scores for each group

Group	Score 1	Score 2	Score 3	Score 4	*P* value (vs. Sham group)
Sham group (*n* = 8)	0	0	1	7	
Hydrogel group (*n* = 8)	0	0	6	2	0.9838
iRoot BP PLUS group (*n* = 12)	0	3	7	2	0.1602
Nar hydrogel group (*n* = 12)	9	3	0	0	< 0.0001

### Nar reduced inflammatory cytokine secretion in dental pulp fibroblasts and neutrophils

**Fig. 4:**
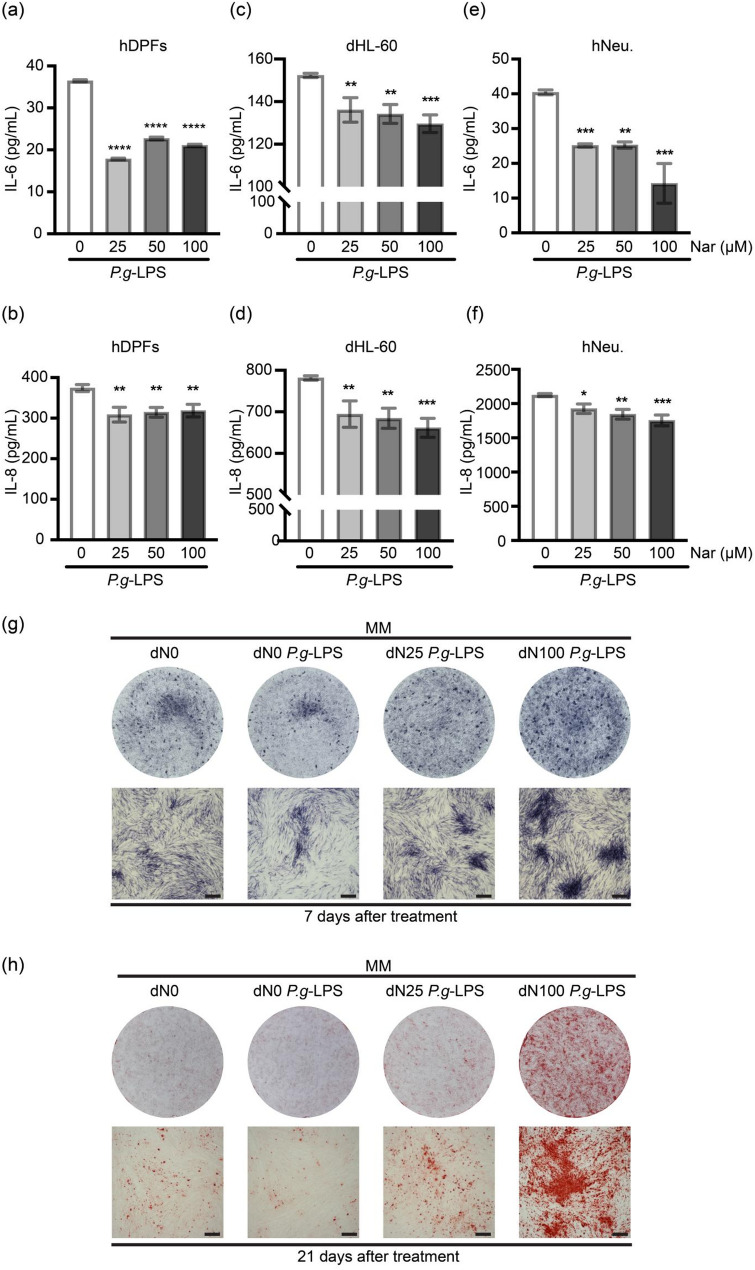
Nar reduced inflammatory cytokine secretion and improved the mineralization of dental pulp fibroblasts. IL-6 and IL-8 quantification in the supernatants of human dental pulp fibroblasts (hDPFs) (**a**, **b**), dHL-60 cells (**c**, **d**), and human peripheral blood-derived neutrophils (hNeu.) (**e**, **f**) induced by *P.g*-LPS for 24 h were measured by ELISA. All results are shown as mean ± SD. ****P < 0.0001,***P < 0.001,**P < 0.01, *P < 0.05. hDPFs were treated with dHL-60 supernatants (untreated or treated with *P.g*-LPS in the absence or presence of Nar for 24 h) combined with MM. **g** Representative images of alkaline phosphatase (ALP) staining after 7-day treatment. **h** Representative images of Alizarin Red S (ARS) staining after 21-day treatment. Scale bars = 500 μm

To investigate the modulatory effects of Nar on human dental pulp fibroblasts (hDPFs) in vitro, the hDPFs, a predominant cell population in dental pulp [18], were isolated, and the FACS analysis showed that hDPFs expressed CD44 (99.9%), CD105 (99.6%), CD90 (99.9%), CD146 (97.8%), vimentin (positive), STRO-1 (1.29%), CD34 (0.41%) and CD45 (0.022%) (Supplementary Fig. S2a, [see Additional file 1]). hDPFs also exhibited increased ALP activity and form mineralized nodules after induction (Supplementary Fig. S2b, c, [see Additional file 1]). Nar at concentrations ranging from 0 to 100 µmol/L did not affect hDPF viability (Supplementary Fig. S2d, [see Additional file 1]). Nar alone did not induce detectable IL-6 or IL-8 secretion in hDPFs; however, it significantly reduced *P.g*-LPS-induced secretion of these cytokines (Fig. 4a, b). Similarly, the inflammatory modulation of Nar was evaluated in neutrophils. Differentiated HL-60 neutrophil-like cells (dHL-60), commonly used as a model for human neutrophils [39], showed CD11b expression by FACS (Supplementary Fig. S2e, [see Additional file 1]). Nar treatment at concentrations up to 100 µmol/L had no detrimental effects on dHL-60 viability (Supplementary Fig. S2f, [see Additional file 1]). After *P.g*-LPS stimulation, Nar significantly reduced IL-6 and IL-8 release from dHL-60 cells (Fig. 4c, d). These findings were further validated in human peripheral blood-derived neutrophils (hNeu.), which were isolated with a purity of 99.4% (CD45⁺CD16⁺CD66b⁺) (Supplementary Fig. S2g, [see Additional file 1]). Nar treatment significantly reduced inflammatory cytokine secretion in human neutrophils as well (Fig. 4e, f). These results suggest that Nar modulates inflammatory responses in both hDPFs and neutrophils in vitro.

### Nar-treated dHL-60 supernatants improved the mineralization of dental pulp fibroblasts inhibited by *P.g*-LPS-treated dHL-60 supernatants

To explore the interaction between neutrophils and hDPF mineralization, the impact of supernatants derived from *P.g*-LPS-treated dHL-60 cells was assessed. Supernatants from *P.g*-LPS-activated dHL-60 cells reduced the ALP expression and mineralization of hDPFs (Fig. 4g, h), suggesting an inhibitory effect of activated neutrophil-like cells on hDPF mineralization. In contrast, supernatants from dHL-60 cells treated with both *P.g*-LPS and Nar restored this change (Fig. 4g, h), indicating that Nar modulates the influence of activated neutrophil-like cells on hDPFs. These findings suggest a poteintal regulatory interaction between hDPFs and neutrophil-derived factors in the inflamed pulp microenvironment.

### Nar promoted the mineralization of dental pulp fibroblasts

**Fig. 5:**
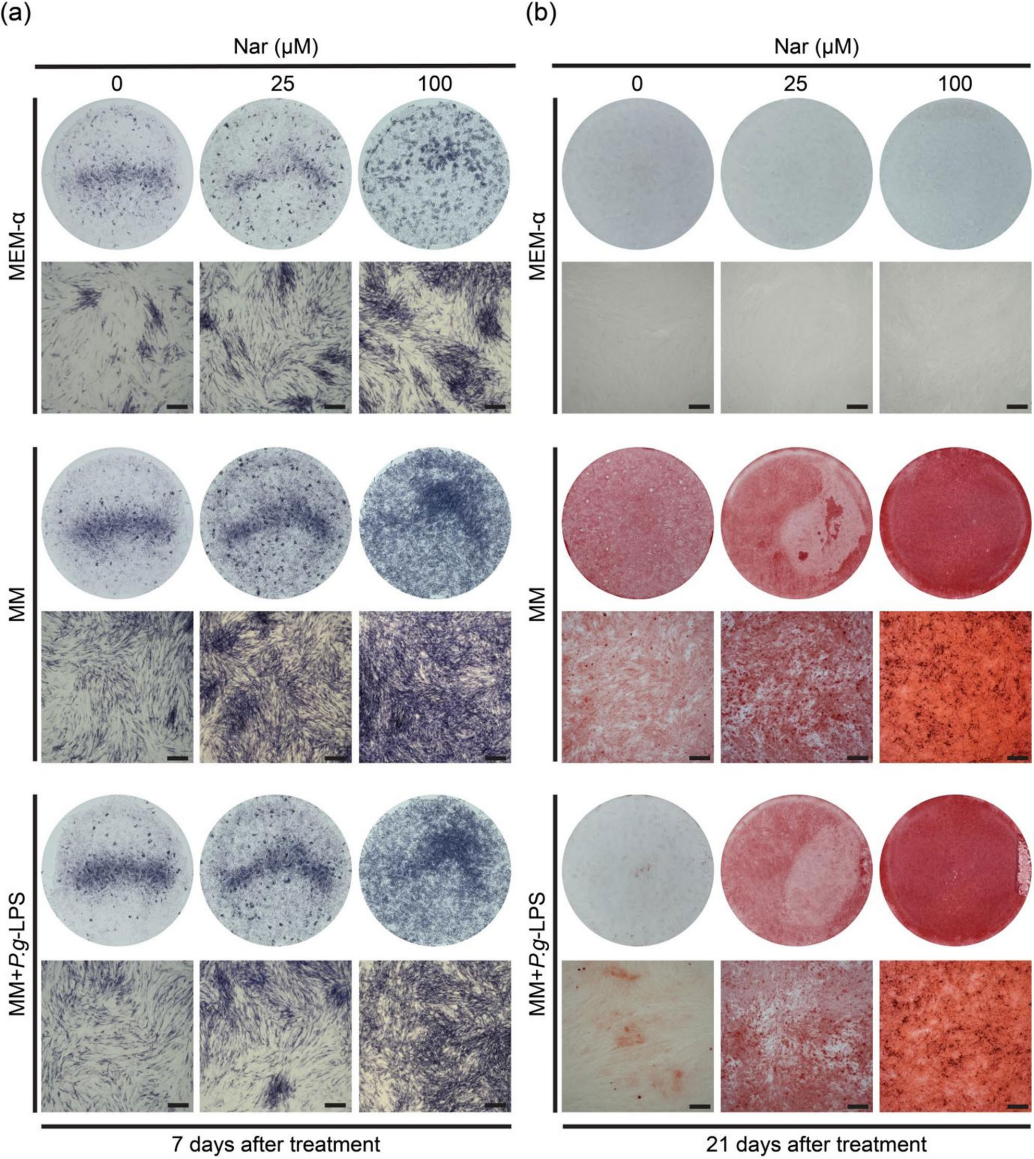
Nar promoted the mineralization of dental pulp fibroblasts. hDPFs were cultured in MEM-α (regular medium), mineralization-inducing medium (MM), or MM supplemented with *P.g*-LPS, with or without Nar. **a** Representative images of alkaline phosphatase (ALP) staining post 7-day treatment. **b** Representative images of Alizarin Red S (ARS) staining post 21-day treatment. Scale bars = 500 μm

The direct effect of Nar on hDPF mineralization was evaluated. After 7 days of treatment, ALP staining showed that Nar increased ALP expression in hDPFs in a dose-dependent manner under MEM-α, mineralization-inducing media (MM), and MM plus *P.g*-LPS culture conditions (Fig. 5a). After 21 days of culture, Alizarin Red S staining showed that Nar had no significant effect on mineralized nodule formation in hDPFs cultured in MEM-α. However, under MM or MM plus *P.g*-LPS conditions, Nar increased mineralized nodule formation in a dose-dependent manner (Fig. 5b). *P.g*-LPS reduced mineralized nodule formation in the MM condition, whereas Nar treatment restored this change (Fig. 5b). These results suggest that Nar enhances ALP expression and hDPF mineralization under mineralization-inducing conditions. In addition, *P.g*-LPS showed minimal effect on ALP expression but was associated with reduced mineralization in hDPFs.

### Nar enhanced the phagocytic and bactericidal capabilities of dHL-60 cells through lysosomal activity

**Fig. 6:**
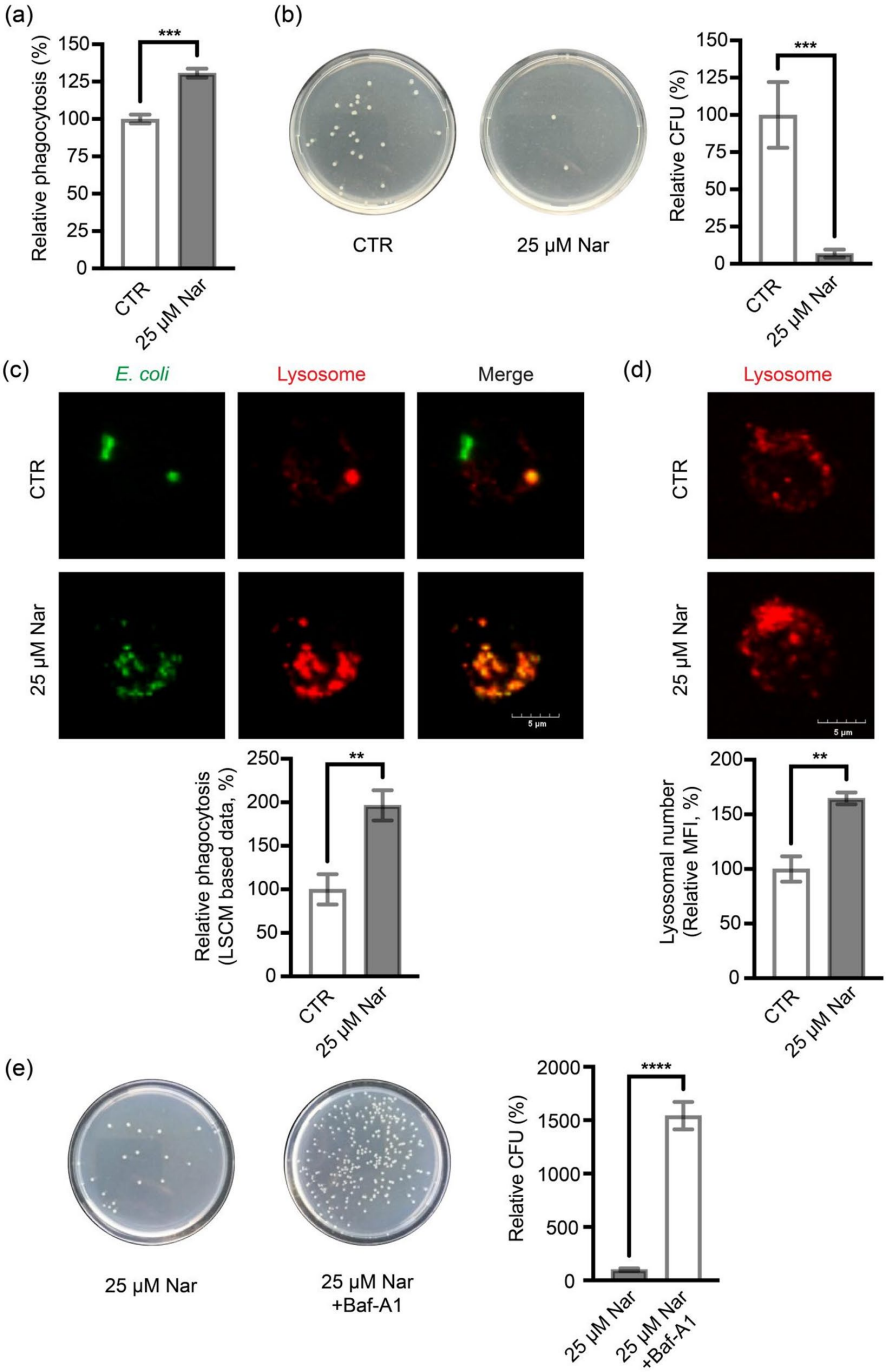
Nar enhanced the phagocytic and bactericidal capabilities of dHL-60 cells through lysosomal activity. Phagocytosis (**a**) and bactericidal activity (**b**) of dHL-60 cells in control (CTR) and Nar-treated groups. Colocalization of lysosomes with EGFP-labeled *E. coli* (**c**), and lysosomal number (**d**) were assessed by LSCM; scale bars: 5 μm. **e** Bactericidal activity by Nar-treated dHL-60 cells in the absence or presence of bafilomycin A1 (Baf-A1), a lysosomal inhibitor. Data are shown as mean ± SD. ****P < 0.0001,***P < 0.001, **P < 0.01

To explore the antimicrobial effects of Nar, its effects on the phagocytosis and bactericidal activity of dHL-60 were examined in vitro. Nar-treated dHL-60 cells engulfed more EGFP-labelled *E. coli* (Fig. 6a) and showed increased bacterial killing (Fig. 6b). Given that lysosomes are responsible for bacterial digestion following phagocytosis, we hypothesized that the increased phagocytic and bactericidal activities of dHL-60 cells may be associated with elevated lysosomal activity. Laser scanning confocal microscope (LSCM) showed increased colocalization of EGFP-labelled *E. coli* with lysosomes in Nar-treated dHL-60 cells (Fig. 6c). In addition, Nar was associated with increased lysosomal fluorescence signals in dHL-60 cells (Fig. 6d). Treatment with bafilomycin A1 (Baf-A1), a widely used lysosomal inhibitor, attenuated the Nar-associated digestion of phagocytosed bacteria (Fig. 6e). These findings support a potential role of lysosomal activity in the bactericidal effects observed with Nar treatment.

### Nar was associated with TFEB-mediated lysosomal activation in dHL-60 cells accompanied by increased intracellular ROS

**Fig. 7:**
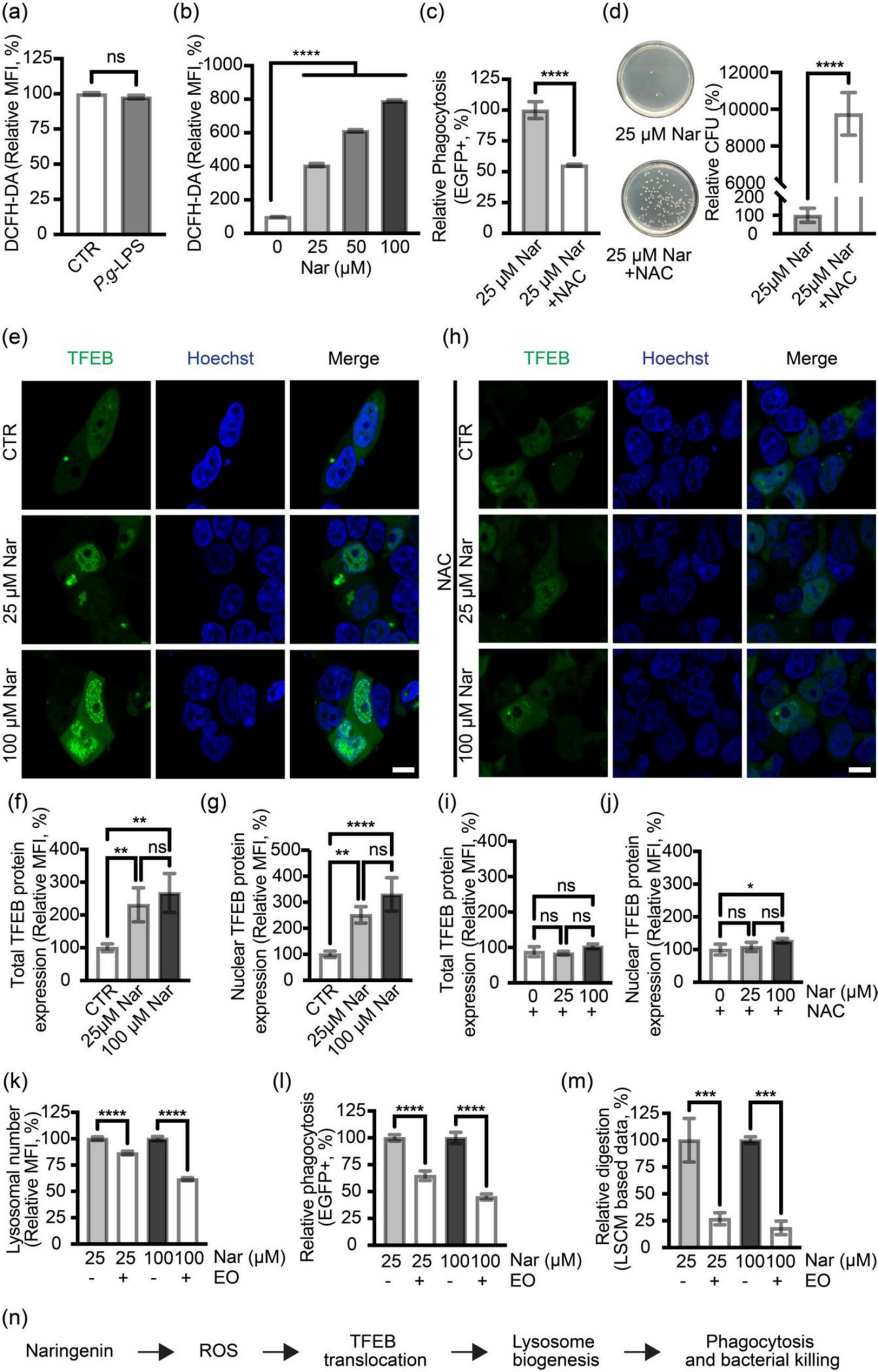
Nar was associated with TFEB-mediated lysosomal activation in dHL-60 cells accompanied by increased intracellular ROS. Intracellular ROS levels were assessed by FACS using DCFH-DA staining. **a** Comparison between untreated control (CTR) and *P.g*-LPS-treated groups. **b** ROS levels in Nar-treated groups. Phagocytosis (**c**) and bactericidal activity (**d**) of dHL-60 cells treated with Nar were evaluated in the absence or presence of N-acetylcysteine (NAC, a ROS scavenger). **e-j** 293T cells transfected with pEGFP-N1-TFEB plasmids were treated with Nar in the absence or presence of NAC. TFEB nuclear translocation was analyzed by LSCM. **e**, **h** Representative LSCM images; scale bars: 10 μm. **f**, **i** Semi-quantification of total TFEB protein expression. **g**, **j** Semi-quantification of nuclear TFEB protein expression. **k** Lysosomal number in dHL-60 cells treated with Nar, in the absence or presence of eltrombopag (EO, a TFEB inhibitor), was detected by FACS. Phagocytosis (**l**) and bacterial digestion (**m**) under the same treatments were assessed by LSCM. **n** Proposed mechanism of Nar-mediated lysosomal activation based on the present study. Data are shown as mean ± SD. ****P < 0.0001, ***P < 0.001, **P < 0.01, *P < 0.05, ns, not significance

TFEB is a key transcriptional regulator of lysosomal biogenesis [40]. External stimulation usually elevates intracellular ROS levels [41], and ROS-induced TFEB activation has been reported to modulate lysosomal number and function [40, 42]. To examine this pathway in dHL-60 cells, DCFH-DA was used as a fluorescent probe to measure intracellular ROS following exposure to *P.g*-LPS or Nar. *P.g*-LPS treatment showed no significant increase in ROS levels (Fig. 7a). However, Nar at concentrations of 0-100 µmol/L induced a dose-dependent increase in intracellular ROS (Fig. 7b). Pretreatment with N-acetylcysteine (NAC), a widely used ROS scavenger, diminished Nar-associated phagocytosis and bactericidal activity of dHL-60 cells (Fig. 7c, d), and attenuated TFEB nuclear translocation (Fig. 7e-j). Eltrombopag (EO), an FDA-approved compound reported to inhibit TFEB by disrupting TFEB-DNA interaction [43], was further used to assess TFEB involvement. EO pretreatment reduced Nar-associated lysosomal biogenesis and diminished Nar-associated phagocytosis and bacterial digestion in dHL-60 cells (Fig. 7k-m). These findings suggest that Nar-induced intracellular ROS is associated with TFEB nuclear translocation, accompanied by increased lysosomal biogenesis and enhanced phagocytosis and bactericidal activity in dHL-60 cells (Fig. 7n).

## Discussion

According to the European Society of Endodontology, preserving and maintaining the pulp vitality is central to contemporary clinical endodontics [5]. Our study suggests that Nar hydrogel may exert beneficial effects in a swine model of pulpitis, as indicated by reduced inflammatory infiltration, the presence of odontoblast-like cells, and formation of dentin-like tissue. The odontoblast-like cells exhibited basally polarized nuclei and DSPP-positive staining, forming a well-organized columnar layer beneath the newly formed dentin. These morphological features align with established criteria for odontoblast differentiation as described in the literature [44].

In our pilot study, iRoot BP PLUS—a widely utilized pulp capping material—served as the positive control [45]. While iRoot BP PLUS promoted mineralized tissue formation in pulp tissue, it did not appear to substantially reduce the inflammatory response. Although an initial inflammatory response is instrumental in pathogen clearance and tissue repair, prolonged or chronic inflammation has been associated with tissue damage and dysregulated mineralization [1, 46]. As demonstrated in the seminal study by Kakehashi et al. [47], exposing the pulp to sterile conditions enables spontaneous dentin bridge formation and preservation of underlying pulp tissue, underscoring the pulp’s intrinsic reparative and mineralization capacity. Conversely, excessive inflammatory stimulation hampers normal dentin formation and compromises pulp tissue integrity. Pulp healing is highly dependent on an intact microcirculation, as adequate vascular perfusion ensures oxygen and nutrient supply as well as clearance of inflammatory by-products [48]. Therefore, inflammation-associated vascular disturbances may further exacerbate hypoxia and impede organized pulp repair.

A distinction existed between the iRoot BP PLUS and Nar hydrogel groups. The iRoot BP PLUS group formed a thicker mineralized bridge but exhibited evident inflammation and reduced coronal pulp tissue. Conversely, the Nar hydrogel group generated a continuous dentin-like bridge while maintaining pulp architecture (greater preservation of coronal pulp tissue) with minimal inflammation. These findings suggest that greater bridge thickness does not necessarily equal to better pulp preservation. The more organized reparative pattern of the Nar hydrogel highlights the importance of modulating inflammation for pulp preservation and organized repair. In contrast, iRoot BP PLUS, primarily composed of tricalcium silicate, promotes mineralization through the creation of an alkaline environment and the release of calcium and phosphate ions.

The hydrogel alone showed limited therapeutic effects compared to the sham group, as it serves mainly as a biocompatible carrier with minimal intrinsic effects. This is supported by prior skin regeneration studies [49, 50]; however, its applicability to the dental pulp’s unique microenvironment remains uncertain. The hydrogel’s microstructural characteristics, such as porosity, degradation, and cytokine diffusion, were not directly assessed in this study. Therefore, the interpretation of the hydrogel-alone effect warrants caution, as the underlying mechanisms require further investigation. 

Our in vitro data show that Nar reduces IL-8 secretion from dental pulp fibroblasts (DPFs), which may contribute to limiting neutrophil recruitment and infiltration at early stages of pulpitis. Moreover, Nar’s capacity to suppress IL-8 production in neutrophils suggests a potential role in modulating inflammatory signaling. These findings are consistent with previous reports linking elevated IL-8 to increased pulpal inflammation severity [51].

One important observation is that Nar improved mineralization-related responses under inflammatory conditions. This process is critical for dentin-pulp complex regeneration in pulpitis. Similar to other flavonoids such as epigallocatechin-3-gallate, taxifolin and isonymphaeol B, Nar has been reported to promote the mineralization capability of cells derived from dental pulp or apical papilla under non-inflammatory stimulation [25, 26, 52]. This capability includes ALP expression and mineralized nodule formation. Our study further showed that Nar improved hDPF mineralization under *P.g*-LPS stimulation. This effect may be associated with Nar’s ability to decrease inflammatory cytokines, given that elevated levels of inflammatory cytokines, including TNF-α, IL-6, and IL-1β, impede mineralization [20]. Our study also showed that *P.g*-LPS impaired the final stages of mineralization by inhibiting mineralized nodule formation in hDPFs. However, it had little effect on ALP expression, which is consistent with previous studies [53, 54]. Our in vivo studies also exhibited the formation of a continuous dentin bridge with tubular structures at the pulp exposure site following reduced inflammation. Previous study has shown that Nar enhances odontogenic differentiation in human dental pulp stem cells by upregulating DMP1 and DSPP expression in cells seeded on toothslice scaffolds [25]. Additionally, TFEB, a crucial modulator of lysosomal activity that also regulates odontoblastic differentiation of dental pulp stem cells, is involved in mineralization [55, 56]. These findings provide a preliminary framework for understanding the mineralization-related effects observed in this study. Further investigations are necessary to clarify the underlying mechanisms and to determine the translational relevance of Nar in pulp inflammation.

Our study suggests that Nar increases phagocytic and bactericidal activities of dHL-60 cells, accompanied by an increase in intracellular ROS levels. This finding aligns with observations in dendritic cells, where similar ROS behavior has been noted [21]. Research demonstrates that Nar activates TFEB in macrophages to promote lysosome biogenesis, a crucial step in cellular waste processing and intracellular pathogen control [57, 58]. Moreover, our study showed that inhibition of intracellular ROS, TFEB nuclear translocation, or lysosomal activity attenuated the bactericidal activity induced by Nar in dHL-60 cells. Specifically, N-acetylcysteine (NAC), eltrombopag (EO), and bafilomycin A1 (Baf-A1) were used to inhibit ROS, TFEB nuclear translocation, and lysosomal activity, respectively. These observations suggest that the bactericidal effect of Nar may involve coordination of intracellular ROS and TFEB-mediated lysosome biogenesis. However, the mechanisms through which Nar-induced ROS results in TFEB activation remain elusive and are a critical area for future research. Together, these findings provide preliminary mechanistic insight into the immunomodulatory effects of Nar on innate immune responses. Nevertheless, the association between enhanced neutrophil bactericidal activity and in vivo pulp healing remains indirect and was not directly examined in the present study.

Limitations of this study include the need to further clarify how Nar modulates inflammation, immune responses, and mineralization, as the detailed molecular pathways remain to be fully understood. Additionally, Nar’s reported ability to alleviate smooth muscle spasms [59] suggests it might affect pulpal vascular function and blood supply; however, this aspect was not directly evaluated in the present study and requires further investigation. Given the complexity of pulp inflammatory and reparative processes, more comprehensive studies are needed to clarify the Nar’s therapeutic role in pulpitis.

## Conclusions

Nar effectively attenuated pulp inflammation, promoted dentin-like tissue formation, and preserved coronal pulp tissue in a swine model of pulpitis. In vitro, Nar reduced inflammatory cytokine release, improved DPF mineralization capability, and modulated neutrophil-related functions. These preclinical findings suggest that Nar may serve as an immunomodulatory adjunct for vital pulp preservation. Further studies are necessary to strengthen the mechanistic understanding of Nar-mediated immunomodulation in pulp inflammation.

## Supplementary Information

Additional file 1. Supplementary antibody information, figures, and tables.

## Data Availability

The data that support the findings of this study are available from the corresponding author upon reasonable request.

## Abbreviations

ALP: Alkaline phosphatase
ARS: Alizarin Red S
Baf-A1: Bafilomycin A1
BSA: Bovine serum albumin
CTR: Control
D: Dentin
DCFH-DA: 2’,7’-dichlorofluorescin diacetate
DCs: Dendritic cells
dHL-60: Differentiated HL-60 cells
DMP1: Dentin matrix protein 1
DMSO: Dimethyl sulfoxide
DPFs: Dental pulp fibroblasts
DSPP: Dentin sialophosphoprotein
EO: Eltrombopag
EGFP: Enhanced green fluorescent protein
FACS: Fluorescence-activated cell sorting
FBS: Fetal bovine serum
Fib.: Dental pulp fibroblasts
GIC: Glass ionomer cement
hDPFs: Human dental pulp fibroblasts
hNeu.: Human peripheral blood-derived neutrophils
H&E: Hematoxylin and eosin
HMGB1: High mobility group box 1
ICC: Immunocytochemical
IHC: Immunohistochemical
IL-1β: Interleukin-1 beta
IL-6: Interleukin-6
IL-8: Interleukin-8
IMDM: Iscove’s Modified Dulbecco’s Medium
IP: Inflamed pulp
LSCM: Laser scanning confocal microscope
MACS: Magnetic-activated cell sorting
MEM-α: α-minimal essential medium
MFI: Mean fluorescence intensity
MM: Mineralization-inducing medium
MN: Mineralized nodules
MPO: Myeloperoxidase
NAC: N-acetylcysteine
Nar: Naringenin
NMT: Newly formed mineralized tissue
Neu.: Neutrophils
NP: Normal pulp
OdLC: Odontoblast-like cells
OcLC: Odontoclast-like cells
*P.g*-LPS: *Porphyromonas gingivalis* lipopolysaccharide
ROS: Reactive oxygen species
SD: Standard deviation
TFEB: Transcription factor EB
TDLT: Tubular dentin-like tissue
TNF-α: Tumor Necrosis Factor-alpha
