# Supplementary material for: Naringenin ameliorates swine pulpitis by modulating immune response
Source: BMC Oral Health. 2026 Apr 14;26:983. doi: 10.1186/s12903-026-08296-5 (PMC13248416; doi:10.1186/s12903-026-08296-5)
Supplement: Supplementary file 1 — Additional file 1. Supplementary antibody information, figures, and tables. [file 12903_2026_8296_MOESM1_ESM.pdf]

Title page

Naringenin ameliorates swine pulpitis by modulating immune response

Qian Wang[1,2], Wenfeng Zeng[3], Huilin Liang[4], Syngcuk Kim[5],

Lanting Shao[1], Yan Yan[1], Yumeng Guo[1], Siqi Huang[1], Ying Zheng[2,*]

[1]School of Stomatology, Capital Medical University, Beijing, China

[2]Department of Stomatology, Peking Union Medical College Hospital, Chinese Academy of Medical Sciences & Peking Union Medical College, Beijing, China

[3]Key Laboratory of Biomacromolecules, Institute of Biophysics, Chinese Academy of Sciences, Beijing, China

[4]Department of Pharmacy, China Pharmaceutical University, Nanjing, China

[5]Department of Endodontics, School of Dental Medicine, University of Pennsylvania, Philadelphia, Pennsylvania, USA

Qian Wang (wangqiangzkg@foxmail.com)

Wenfeng Zeng (zengwenfeng@ibp.ac.cn)

Huilin Liang (2020210170@stu.cpu.edu.cn)

Syngcuk Kim (syngcuk@pobox.upenn.edu)

Lanting Shao (slt2021@mail.ccmu.edu.cn)

Yan Yan (yanandf3enc@163.com)

Yumeng Guo (ymguo@mail.ccmu.edu.cn)

Siqi Huang (huangsiqi1003@mail.ccmu.edu.cn)

***Corresponding author:** Ying Zheng, Department of Stomatology, Peking Union Medical College Hospital, Chinese Academy of Medical Sciences & Peking Union Medical College, Beijing, China. Tel: +0086-10-69151742, Fax: 010-83911316. Email: zhengying49@pumch.cn

Additional file 1

Antibodies

The primary antibodies were used in this study include: rabbit anti-MPO polyclonal antibody (22225-1-AP, Proteintech) at a dilution of 1:200; rabbit anti-HMGB1 polyclonal antibody (10829-1-AP, Proteintech) at 1:200; mouse anti-DSPP monoclonal antibody (sc-73632, Santa Cruz Biotechnology) at 1:200; and rabbit anti-vimentin polyclonal antibody (GB11192, Servicebio) at 1:500.

Secondary antibodies include: HRP-conjugated goat anti-mouse IgG (H+L) antibody (31430, Invitrogen) and HRP-conjugated goat anti-rabbit IgG (H+L) antibody (GB23303, Servicebio) were applied at a 1:200 dilution respectively.

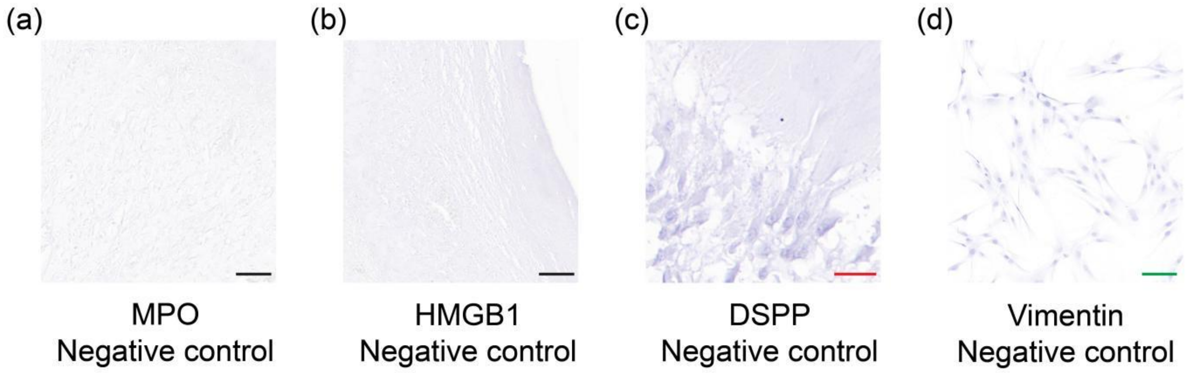

Supplementary Fig. S1 Negative controls for immunohistochemistry and immunocytochemistry. Representative negative control images obtained by omitting the primary antibody for **(a)** MPO, **(b)** HMGB1, and **(c)** DSPP immunohistochemical staining in swine pulp tissue, and **(d)** vimentin immunocytochemical staining in dental pulp fibroblasts. No specific staining was observed in any control samples. Scale bars: 100 μm (green), 50 μm (black), 20 μm (red).

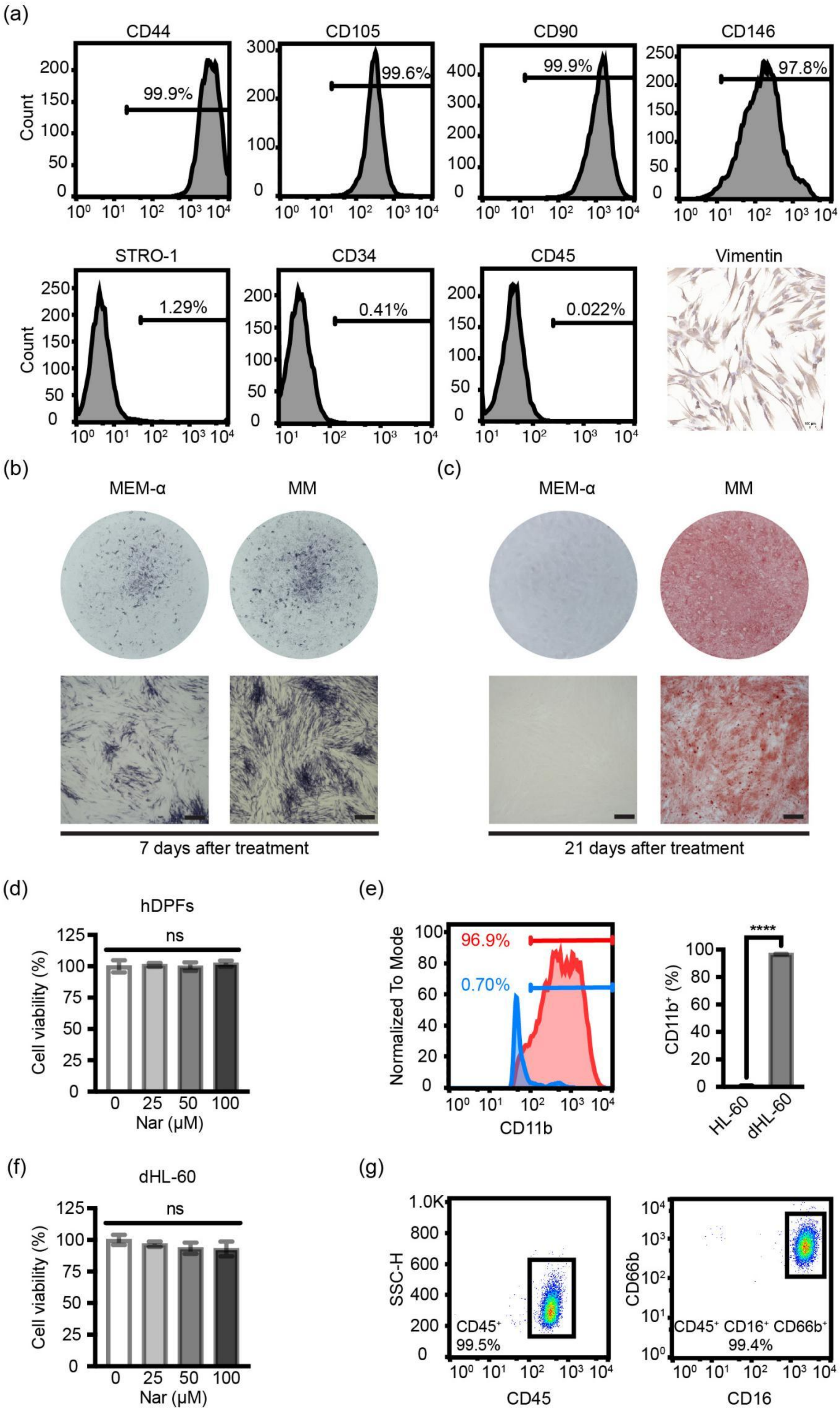
(a)
CD44
99.9%
CD105
99.6%
CD90
99.9%
CD146
97.8%
Count
STRO-1
1.29%
CD34
0.41%
CD45
0.022%
Vimentin
(b)
MEM-α
MM
7 days after treatment
(c)
MEM-α
MM
21 days after treatment
(d)
hDPFs
ns
Cell viability (%)
Nar (μM)
(e)
Normalized To Mode
96.9%
0.70%
CD11b
****
CD11b⁺ (%)
HL-60
dHL-60
(f)
dHL-60
ns
Cell viability (%)
Nar (μM)
(g)
SSC-H
CD45⁺
99.5%
CD45
CD66b
CD45⁺ CD16⁺ CD66b⁺
99.4%
CD16

Supplementary Fig. S2 Cell preparation and identification of human dental pulp fibroblasts (hDPFs), differentiated HL-60 neutrophil-like cells (dHL-60), and human peripheral blood-derived neutrophils (hNeu.). **(a)** Purified hDPFs were identified through the FACS and ICC analyses. **(b)** Representative images of alkaline phosphatase (ALP) staining after 7-day treatment. **(c)** Representative images of Alizarin Red S (ARS) staining post 21-day treatment. Scale bars = 500 μm. **(d)** CCK8 assays were used to assess the viability of hDPFs after treatment with 0-100 μmol/L Nar for 24 hours. **(e)** dHL-60 cells were induced with DMSO for 7 days, and identified using the FACS analysis. **(f)** CCK8 assays were used to assess the viability of dHL-60 cells after treatment with 0-100 μmol/L Nar for 24 hours. **(g)** Isolated hNeu. were identified through the FACS analysis. All results are shown as mean ± SD. *****$P < 0.0001$*; ns indicates no significant difference.

Supplementary Table. S1 List of antibodies used for fluorescence-activated cell sorting (FACS) analysis.

**Antibody**	**Clone number**	**Catalog number**	**Conjugate**	**Source**
CD44 mAb	IM7	12-0441-81	PE	eBioscience
CD105 (Endoglin) mAb	SN6	12-1057-41	PE	eBioscience
CD90 (Thy-1) mAb	eBio5E10 (5E10)	17-0909-41	APC	eBioscience
CD146 mAb	P1H12	12-1469-41	PE	eBioscience
STRO-1 mAb	STRO-1	PE-65184	PE	ProteinTech
CD34 mAb	QBEnd-10	FITC-65183	FITC	ProteinTech
CD45 mAb	HI30	11-0459-41	FITC	eBioscience
CD16 mAb	eBioCB16 (CB16)	12-0168-41	PE	eBioscience
CD66b mAb	G10F5	17-0666-42	APC	eBioscience
CD11b mAb	ICRF44	17-0118-41	APC	eBioscience
